# Metabolic Profiling in Human Fibroblasts Enables Subtype Clustering in Glycogen Storage Disease

**DOI:** 10.3389/fendo.2020.579981

**Published:** 2020-11-23

**Authors:** Luciana Hannibal, Jule Theimer, Victoria Wingert, Katharina Klotz, Iris Bierschenk, Roland Nitschke, Ute Spiekerkoetter, Sarah C. Grünert

**Affiliations:** ^1^ Laboratory of Clinical Biochemistry and Metabolism, Department of General Pediatrics, Adolescent Medicine and Neonatology, Faculty of Medicine, Medical Center—University of Freiburg, Freiburg, Germany; ^2^ Life Imaging Center, Center for Integrated Signalling Analysis, Albert-Ludwigs-University, Freiburg, Germany; ^3^ BIOSS Centre for Biological Signaling Studies, Albert-Ludwigs-University Freiburg, Freiburg, Germany; ^4^ Department of General Pediatrics, Adolescent Medicine and Neonatology, Faculty of Medicine, Medical Center—University of Freiburg, Freiburg, Germany

**Keywords:** inborn error of metabolism, metabolism, metabolomics, mitochondria, glycogen storage disease, energy deficiency, redox homeostasis

## Abstract

Glycogen storage disease subtypes I and III (GSD I and GSD III) are monogenic inherited disorders of metabolism that disrupt glycogen metabolism. Unavailability of glucose in GSD I and induction of gluconeogenesis in GSD III modify energy sources and possibly, mitochondrial function. Abnormal mitochondrial structure and function were described in mice with GSD Ia, yet significantly less research is available in human cells and ketotic forms of the disease. We hypothesized that impaired glycogen storage results in distinct metabolic phenotypes in the extra- and intracellular compartments that may contribute to pathogenesis. Herein, we examined mitochondrial organization in live cells by spinning-disk confocal microscopy and profiled extra- and intracellular metabolites by targeted LC-MS/MS in cultured fibroblasts from healthy controls and from patients with GSD Ia, GSD Ib, and GSD III. Results from live imaging revealed that mitochondrial content and network morphology of GSD cells are comparable to that of healthy controls. Likewise, healthy controls and GSD cells exhibited comparable basal oxygen consumption rates. Targeted metabolomics followed by principal component analysis (PCA) and hierarchical clustering (HC) uncovered metabolically distinct poises of healthy controls and GSD subtypes. Assessment of individual metabolites recapitulated dysfunctional energy production (glycolysis, Krebs cycle, succinate), reduced creatinine export in GSD Ia and GSD III, and reduced antioxidant defense of the cysteine and glutathione systems. Our study serves as proof-of-concept that extra- and intracellular metabolite profiles distinguish glycogen storage disease subtypes from healthy controls. We posit that metabolite profiles provide hints to disease mechanisms as well as to nutritional and pharmacological elements that may optimize current treatment strategies.

## Introduction 

Glycogen storage diseases (GSDs) are a group of inborn errors of metabolism that result from defects in any of the enzymes involved either in glycogen synthesis or glycogen degradation (1, 2). GSDs can be divided in two major entities: GSDs with hepatic involvement that usually present with hypoglycemia and often hepatomegaly, and muscular GSDs with predominantly neuromuscular symptoms such as muscle weakness and/or hypotonia (3). The classical liver GSDs comprise type I, III, VI, IX, and 0. While all other subtypes are characterised by hepatic glycogen storage due to either impaired glycogen breakdown, or the hydrolysis (GSD Ia) or transport of glucose-6-phosphate (GSD Ib), in GSD 0 the synthesis of glycogen within the hepatocyte is impaired. Several cellular mechanisms that contribute to the pathogenesis of these diseases have been investigated, however, little is known about the function of different organelles in these disorders, with only one comprehensive study performed in mice with GSD Ia examining mitochondria in hepatocytes (4) and one study assessing mitochondrial complexes activities in human lymphocytes (5).

We herein investigate GSDs types Ia, Ib and III. Glycogen storage disease type Ia, also known as von Gierke disease (6) is caused by mutations in the *G6PC* gene (OMIM 613742) (7) encoding glucose-6-phosphatase. The disease manifests early in life with severe hypoglycemia and hepatomegaly due to excessive storage of glycogen in the liver (7, 8). Glycogen storage disease type Ib is a second subtype of von Gierke disease caused by mutations in the *G6PT1* gene (*SLC374A*, OMIM 602671) (9), which encodes the glucose-6-phosphate transporter. In GSD Ib, the enzyme glucose-6-phosphatase is active but the transporter defect results in a functional deficiency of glucose-6-phosphate that impedes the liberation of glucose (10). The major clinical manifestations of GSD Ia and Ib include recurrent hypoglycaemia, lactic acidosis, hyperlipidemia, and hepatomegaly. Patients with GSDIb are prone to infections due to neutropenia and neutrophil dysfunction and often present with inflammatory bowel disease (11, 12). Glycogen storage disease type III is caused by mutations in the *AGL* gene (OMIM 610860) (13, 14) that encodes a glycogen debranching enzyme that possesses two catalytic activities, namely, amylo-1,6-glucosidase, and 4-alpha-glucanotransferase. Patients with GSD III present clinical manifestations in infancy that include hypoglycaemia, hepatomegaly and growth delay (13). The disorder has been classified in subtypes according to the organ-specific enzyme deficiency (15, 16). Patients with both liver and muscle involvement belong to GSD IIIa (17), the most common subtype, whereas patients having exclusive liver involvement belong to GSD IIIb.

Glycogen storage diseases impair glycogen synthesis, breakdown, and transport of products thereby disturbing carbohydrate homeostasis in the cell (3). Because carbohydrates are major fuels for cellular energy, previous studies aimed to identify the role of mitochondria and other energy producing pathways in the pathogenesis of GSD. Studies with animal models revealed reduced mitochondrial content, abnormal ultrastructure of cristae and overall morphology, and altered mitophagy in the liver of GSD Ia (4, 18). Analysis of Krebs cycle metabolites in animal liver showed a reduction of succinate in GSD Ia but no other remarkable findings in this pathway of energy production compared to healthy controls (4). Another study in mice identified downregulation of sirtuin 1 signaling in GSD Ia as a possible mediator of mitochondrial function (19). The authors proposed that this impairment in sirtuin 1 signaling contributes to the development of hepatocellular adenoma/carcinoma in GSD Ia (19). Investigation of metabolic stress induced by diet in a liver-specific knock-out mouse model of GSD Ia showed that diseased livers reprogram metabolism to a phenotype that resembles cancer cells (20). This included upregulation of glycolysis and *de novo* lipogenesis and a reduction in fatty acid oxidation (20). Further, these metabolic changes were associated with reduced autophagy, antioxidant enzymes and apoptosis, and impaired endoplasmic reticulum stress response (20).

A study by Rossi examined plasma and urine markers of metabolism in patients with GSD Ia versus a cohort of age- and gender-matched controls (21 GSD I patients, 14 GSD Ia and 7 GSD Ib) (21). The study identified greater levels of plasma short-chain acylcarnitines and increased excretion of organic acids in urine in GSD Ia (and to a lesser extent in GSD Ib patients) compared to healthy controls (21). The greater levels of short-chain acylcarnitines were speculated as to possibly represent mitochondrial impairment, but no further testing was performed (21). In another study, associations between reduced bone mineral density and insulin resistance in GSD III were hypothesized to implicate mitochondrial impairment as one of the contributors to the observed osteopenia/osteoporosis, but no studies were performed to corroborate this hypothesis (18).

Evidence of mitochondrial impairment was also provided in a study that measured enzymatic activities in lymphocytes of patients with GSD I, GSD III, GSD VI, and GSD IX (5). Results from this study identified reduced activity of succinate dehydrogenase (complex II of the respiratory chain) and glycerol-3-phosphate dehydrogenase (glycolysis), and increased enzymatic activity of NADH dehydrogenase and lactate dehydrogenase (LDH) in all GSD types, with most pronounced changes in enzymatic activity found in GSD I (5).

While previous studies point to a role of mitochondria in the pathogenesis of GSD Ia in mice, no studies have been performed in the other subtypes of hepatic GSDs or with human cells. This has prompted us to investigate mitochondrial morphology and function in skin fibroblasts from healthy controls, patients with GSD subtypes Ia and GSD Ib (both non-ketotic), and patients with GSD III, representing the most severe of the ketotic liver GSDs. Herein, we employ targeted metabolomics to gather information about energy producing pathways in GSD and to gain insights into underlying pathomechanisms.

Our studies with human skin fibroblasts reveal that: a) Mitochondrial networks, volume and oxygen consumption rates are comparable in healthy controls and GSD types Ia, Ib, and III; b) Principal Component Analysis (PCA) and Hierarchical Clustering (HC) analysis of extracellular and intracellular metabolites permits the clustering of healthy controls versus GSD subtypes suggesting distinct metabolic poise in health and disease; and c) The concentration of metabolites of energy producing pathways (lactate, Krebs cycle intermediates) and antioxidant defense (cysteine, glutathione) suggest dysfunctional electron transfer in the respiratory chain, consistent with impaired mitochondrial function in GSD compared to healthy controls.

## Materials and Methods

### Reagents and Solvents

Culture media, antibiotics, fetal bovine serum (FBS), reagents, solvents and buffers were used as directed by the respective manufacturers without further processing. Water (product Nr. 39253) and methanol (product Nr. 34966) for mass spectrometry were purchased from Honeywell (Riedel-de Haen, Germany). Stable isotopes for metabolomic analysis were purchased from Sigma-Millipore (Merck Group, Germany).

### Primary Skin Fibroblasts

Skin fibroblasts from healthy subjects were used and characterized in two previous studies (22, 23). Healthy control fibroblasts were from donors in the neonatal stage as well as from an 8-year-old male subject (22, 23). Skin fibroblasts from patients with GSD were obtained from the Coriell Institute Repository, New Jersey, USA. The description of the GSD cells utilized in this study and references to previous genetic and/or enzymatic activity characterizations by others are described in **Table 1**.

**Table 1:** Glycogen storage disease dermal fibroblasts employed in this study^1^.

Cell ID	Disease type	Phenotype	Gender	Race/Ethnicity	Age at collection	References
GM00574	GSDIa	Liver biopsy shows absent glucose-6-phosphatase	Male	White	25 years old	(24)
GM03719	GSDIb	Liver biopsy shows G6P transport defect	Male	Panama Indian	5 years old	(10, 11, 24)
GM02523	GSDIII	Decreased activity of amylo-alpha-1,6-glucosidase (debrancher enzyme) in fibroblasts	Male	Black/African American	1 year old	(14)
GM03390	GSDIII	Deficient amylo-1-6-glucosidase activity in fibroblasts	Male	White/Arab	1 year old	(14)
GM03388	GSDIII	Deficient amylo-1-6-glucosidase activity in fibroblasts	Male	White/Iranian	1 year old	Coriell Institute Repository (online)

^1^Information obtained from Coriell Institute Repository and published manuscripts.

### Cell Culture

Skin fibroblasts from healthy subjects and from patients with GSD (passages 4 to 15) were cultured in DMEM low glucose supplemented with GlutaMax (Gibco, product Nr. 21885108; culture medium composition provided in Supporting Information, **Table S1**), 10% FBS, and antibiotic and antimycotics. Cells were fed with fresh medium every 2–3 days, and passaged at a 1:3 ratio by trypsinization and centrifugation. All cells were grown in a humidified 5% CO_2_ incubator at 37°C. Although dermal fibroblasts possess high metabolic activity even in their quiescent state (25) and their suitability as a model to study human metabolism has been assessed in-depth (26), to ensure comparability of mitochondrial turnover and metabolite homeostasis all cell cultures were synchronized by adjusting their seeding density and/or total growing time between passages such that end-point harvesting was carried out at comparable cell density. Cells from patient with GSD Ia grew slightly slower than all other cells, therefore, these cultures were given 2–3 extra days to grow or were seeded at an initially higher cell density. This permitted reproducible pacing of the cell cultures, which were grown in parallel for all experiments described in this work. At time of harvesting, cell cultures from healthy controls and GSD patients exhibited comparable morphology and total cell number.

### Confocal Microscopy

Analysis of mitochondrial organization and volume was performed by spinning-disk confocal microscopy (Axio Observer Z1 with Yokogawa CSUX1FW, camera Photometrics sCMOS PRIME; Carl Zeiss Microscopy GmbH, Germany). Cells were grown under standard conditions (humidified 5% CO_2_ incubator at 37°C) for 3–5 days, trypsinized and 1000 cells were seeded in glass bottom cell culture dishes (Ibidi, Munich, Germany, µ-dish 35 mm diameter, order No. 81218-200). Cells were grown overnight under standard conditions. Live cell staining was applied using MitoView™ 650 (product Nr. 70075-T, Biotium, CA, USA) for mitochondria and Calcein™ AM (product Nr. C1430, Invitrogen, CA, USA) for cell body. Three independent batches of cells at three independent locations were imaged on three different days (one batch per day), for three healthy controls and the five GSD fibroblasts described in **Table 1**. In order to maintain consistency in terms of cell culture age, all cells were seeded 24 h prior to the imaging experiment.

Images from live cells were recorded using the ZEISS ZEN blue software (Version 2.6, Carl Zeiss Microscopy GmbH, Germany) with the following conditions: incubation at 37°C with 5% CO_2_, objective C-Apochromat 63x/1.2 water immersion, pixel size x/y 0.1 µm, excitation 561 nm, emission filter BP 629/62 nm, exposure time 150 ms for MitoView, excitation 488 nm, emission filter BP 525/50 nm, exposure time 50 ms for Calcein. At each location a z-stack image consisting of 9 overlapping tiles (area 314 x 472 µm) and 60 z-planes (distance 0.31 µm) was recorded. Tile images were stitched together and deconvolved with Huygens software (Vers. 19.04, Scientific Volume Imaging, Netherland). The volume of mitochondria was estimated using the surface and statistics module of the software Imaris (V9.5.1 Bitplane AG, Switzerland).

### Determination of Oxygen Consumption Rates

Oxygen consumption rates were determined with a cell-impermeable phosphorescent oxygen probe (product Nr. 600800, Cayman, Michigan, USA). 96-well plates were seeded at a density of 50,000 cells per well. The cells were grown for 18 h in standard DMEM supplemented with 10% FBS. Measurement of fluorescence was performed with excitation set at 380 nm and emission at 650 nm, a delay time of 30 µs and a read time of 100 µs on a Tecan Infinite Pro plate reader set at 37°C. All samples were run in triplicates. Results from this measurement are shown in **Figure S1**.

### Determination of Lactate Using the LDH Reaction

Lactate is a marker metabolite of glycolytic activity that undergoes export into the extracellular medium. Production of lactate was monitored by two separate methods. Firstly, a colorimetric detection of lactate was carried out enzymatically *via* de reaction of lactate dehydrogenase (LDH) in a sample of conditioned culture medium (product Nr. 600450, Cayman, Michigan, USA). Cells were seeded at a density of 30,000 cells per well. The cells were grown for 4 h in standard DMEM supplemented with 10% FBS (to enable cell attachment), and the medium was then replaced with DMEM supplemented with 1% FBS (reduction of FBS concentration minimizes the contribution of LDH in FBS). The assay was performed as directed by the manufacturer. Quantification was performed with a calibration curve with pure lactate 0–10 mM. All samples were run in triplicates. Secondly, lactate was identified and quantified by liquid chromatography mass spectrometry using a targeted metabolomic method for organic acids (please, see section *Targeted Analysis of Metabolites by Liquid Chromatography Tandem Mass Spectrometry* below). Results of lactate concentration determined from colorimetric and LC-MS/MS methods are shown in **Figure S2A** (colorimetric) and **Figure S2B**, LC-MS/MS).

### Preparation of Cell Pellets and Collection of Conditioned Culture Medium for Metabolomics

Cells were cultured in T25 flasks (three flasks for each of healthy control and GSD fibroblasts) for 5 days without medium exchange, harvested by trypsinization, and washed once with PBS by centrifugation. Dry cell pellets were frozen in dry ice and stored at -80°C for further processing, as described (22). On the day of cell harvesting (on day 5 after no media exchange), conditioned culture medium was collected and centrifuged at 9847 *x g* for 10 min to remove dead cells and debris. Cleared conditioned medium was transferred into a clean 1.5 ml Eppendorf microcentrifuge tube and stored at -80°C for further processing, as described (22).

### Targeted Analysis of Metabolites by Liquid Chromatography Tandem Mass Spectrometry

Metabolites of the methionine cycle, trans-sulfuration and glutathione pathway in their thioether, oxidized and reduced forms, creatinine, S-adenosylmethionine, and S-adenosylhomocysteine were determined according to a published procedure (22). In brief, an aliquot of cell lysate or conditioned medium (20 µl) was mixed with 20 µl of H_2_O (non-reducing conditions) or 20 µl DTT 0.5 M (reducing conditions), vortexed, and incubated at room temperature for 10 minutes. An aliquot of internal standard was added (20 µl) and metabolites were extracted by addition of 100 µl of 0.1% formic acid in MeOH (22). Metabolites of the Krebs cycle and organic acids of special relevance for cell metabolism (lactate, glycolysis) and mitochondrial respiration (malonate, itaconate, methylmalonate) were determined by isotopic dilution, based on the same published procedure (22) with modifications. Briefly, chromatographic conditions were adjusted to an isocratic run of 50% solvent A (0.1% formic acid in water) and 50% solvent B (0.1% formic acid in water). Calibration curves of Krebs cycle intermediates were created in the range 0-500 µM. Internal standards consisted of isotopically labeled analytes, that included the following: D_4_-succinate, D_3_-methylmalonate, ^13^C_5_-itaconate, ^13^C_1_-lactate, and D_3_-malonate. Analytes for which isotopically labeled variants were not available were normalized with respect to D_3_-methylmalonate.

### Data Analysis

Mass spectrometry data was analyzed with the Analyst^®^ software version 1.6 (AB Sciex).

We first examined the data by applying PCA, a multivariate statistical method that captures not only changes in individual metabolites between different groups, but it also assesses dependency between single metabolites (27, 28). PCA is a linear projection of the original data into lower dimension orthogonal axes. The direction of these new axes is optimized to maximize the variance of the projected data points (i.e., spread of the data points). In so doing, PCA guides the identification of metabolite contributions to disease phenotypes. Herein, PCA was performed on metabolite data to reduce data dimensionality and differentiate metabolite profiles across samples. Ellipses were drawn at 95% confidence around the mean of the data points within disease groups.

Samples were also clustered accordingly to their metabolic profiles using HC. HC was also performed to cluster metabolites accordingly to their sample profiles.

Oxygen consumption rates were estimated by fitting a double exponential curve to the measures of oxygen consumption over time.

PCA and HC were carried out in R 3.6.3 (R Core Team, New Zealand). Curve fitting and boxplots were created using MATLAB version R2019b (MathWorks, Natick, USA).

Individual comparisons of absolute metabolite concentrations between healthy controls and GSD disease groups were performed with one-way ANOVA. The significance level was set to α = 0.05.

## Results

### Growth Characteristics of GSD Fibroblasts

**Figure 1:**
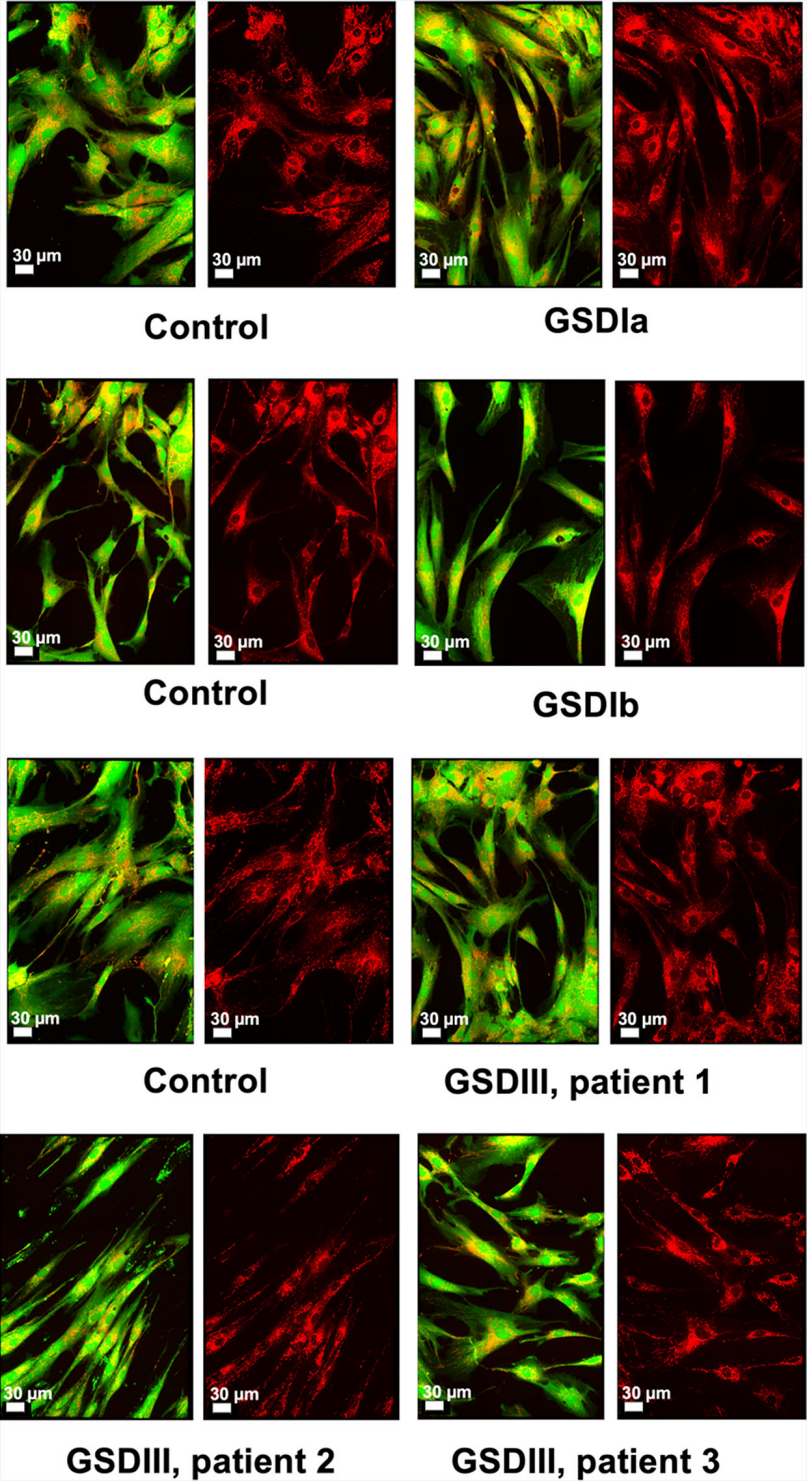
Microscopy of live human skin fibroblasts from healthy controls and GSD patients. Spinning-disk live imaging of cells (green and red overlay) and their mitochondria (right panels, red). The images of three independent healthy controls and the different GSD subtypes revealed high similarity both in cellular morphology and mitochondrial distribution. Cell bodies were stained with Calcein™ AM (Invitrogen) and mitochondria were stained with MitoViewTM 650 (Biotium). Scale shows 30 µm.

Inherited diseases of glycogen metabolism are complex disorders characterized by systemic energy deficiency. In certain GSDs, specific organs such as liver and kidney exhibit structural abnormalities due to excessive glycogen deposition. While human hepatocytes and renal cells from patients with GSD are unavailable for research purposes, a few research laboratories and the Coriell Institute for Medical Research (New Jersey, USA) have possession of dermal fibroblasts from patients with GSD. This precious source of primary cells from patients derives from an era prior to molecular genetic diagnostics, whereby performing skin biopsies was a routine procedure to diagnose patients (29). Herein, we investigated mitochondrial organization and distribution, and energy metabolism in human primary fibroblasts. Cultured cells from healthy subjects and from GSD patients (passages 4 to 15, all from donors ≤ 25 years old at time of skin biopsy) exhibited normal fibroblast morphology (**Figure 1**), in agreement with the literature for the specific GSD cell lines utilized in this study (10, 11, 14, 24). Patient fibroblasts with GSD Ia grew slightly slower compared to healthy controls or the other GSD types, which was paced with the other experimental cell cultures by allowing 2–3 extra days in culture or by seeding cultures of GSD Ia at a greater cell density. Overall, the distribution of mitochondria across the cell body was comparable for healthy controls and all GSD subtypes (**Figure 1**, red staining). Oxygen consumption rates (OCR) were measured under standard cell culture conditions, using a phosphorescent oxygen probe. No differences were observed in basal oxygen consumption rates between healthy control and GSD fibroblasts under our experimental conditions (**Figure S1**).

### Mitochondrial Organization and Volume in GSD Fibroblasts

**Figure 2:**
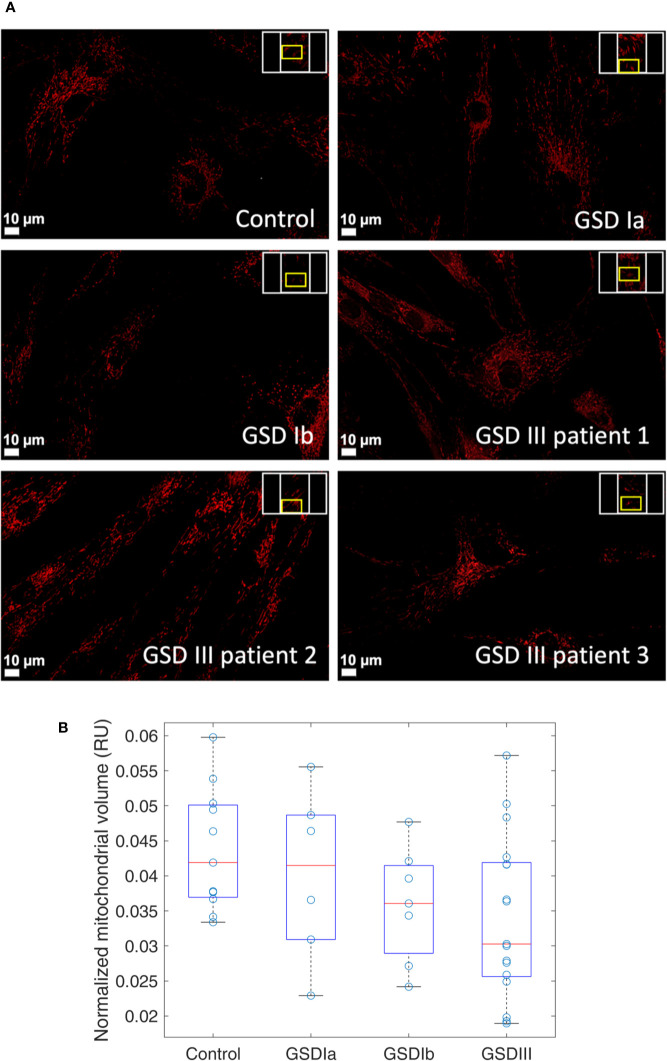
Visualization and quantification of mitochondria in human skin fibroblasts from healthy controls and from GSD patients. Panel **(A)** Spinning-disk live imaging of mitochondria (red, MitoViewTM 650, Biotium). Scale bars show 10 µm. Panel **(B)** Boxplots of relative mitochondrial volume measurements in healthy controls and in patients with GSD Ia, GSD Ib and GSD III. Each data point represents a ratio of mitochondrial volume by the respective cellular surface. This ratio is herein expressed as relative units (RU). A minimum of three regions were examined per sample. In the boxplots, the red lines indicate the median and the box hinges describe the 1st and 3rd quartiles. The box whiskers represent the most extreme data value that is not larger (or smaller) than 1.5 times the interquartile range. Boxplots show a similar median in all the groups with less variability observed in the GSD Ib group. Statistical analysis by Kruskal-Wallis retrieved no significant differences between the controls and GSD subtypes (α = 0.05).

The organization of mitochondria in cultured fibroblasts was examined by spinning-disk confocal microscopy using a live-cell staining protocol that retained the fluorescent dyes for up to 72 h as described in methods. Under our culture conditions, mitochondrial network morphology was comparable between healthy controls and GSD cells (**Figure 2A**), suggesting no marked alterations in organelle turnover by fission and fusion under our experimental conditions. Cloned images showing mitochondrial volume in a tridimensional perspective and also in greyscale are provided in supporting information (**Figure S3**). Results from quantification of relative mitochondrial volume normalized by cell surface showed comparable content of the organelle in healthy controls and GSD groups (**Figure 2B**). A tendency toward slightly lower mitochondrial volumes was seen in all GSD cells, however, these differences did not reach statistical significance (Kruskal-Wallis, α = 0.05).

### Metabolic Profiling of GSD Fibroblasts by Targeted Metabolomics

In order to examine whether disruption of glycogen storage in GSD fibroblasts results in a distinct metabolic poise compared to healthy control fibroblast we performed a targeted metabolomic analysis of cell lysates (intracellular metabolites) and conditioned culture medium (extracellular metabolites). Carbohydrate metabolism fuels major pathways of energy production in cells, so we examined glycolysis end product lactate, Krebs cycle intermediates, the methionine cycle and folate metabolites, the transsulfuration pathway and glutathione metabolism. Previous studies performed with animal models of GSD suggested increased oxidative stress in GSD. Therefore, we herein examined the two major low-molecular antioxidants in cells, namely, cysteine and glutathione in their oxidized and reduced forms, as well as their corresponding precursor metabolites that derive from the methionine cycle and the trans-sulfuration pathway. Because renal injury is a clinical feature of GSD I, we also determined creatinine. A total of 20 extracellular metabolites and 31 intracellular metabolites were measured by LC-MS/MS in a fully quantitative manner.

**Figure 3:**
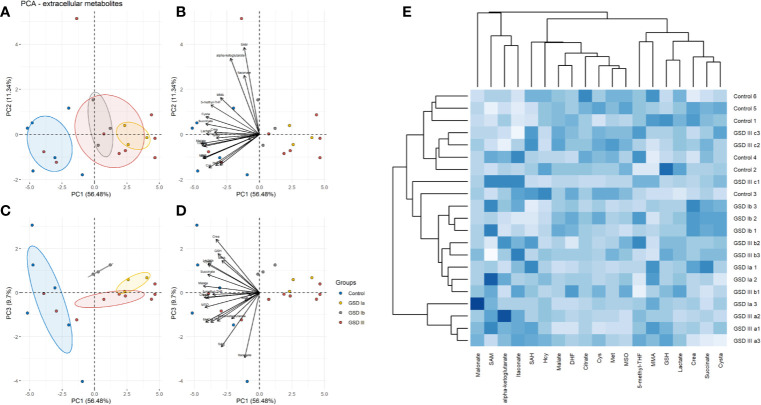
Principal component analysis (PCA) of extracellular metabolites. Panel **(A)** Score plots of PC1-PC2. Panel **(B)** Loading plots of PC1-PC2. Panel **(C)** Score plots of PC1-PC3. Panel **(D)** Loading plots of PC1-PC3. Control: n=3; GSDIa n =1; GSDIb n=1; GSDIII n=3, each by triplicate. A total of 19 metabolites were included in the analysis. Ellipses shown at 95% confidence centered around the mean of data points. The small overlap among ellipses depicts differences between healthy controls and disease subtypes. Panel **(E)** Heatmap of extracellular metabolites with hierarchical clustering (Euclidean distance, complete linkage).

**Figure 4:**
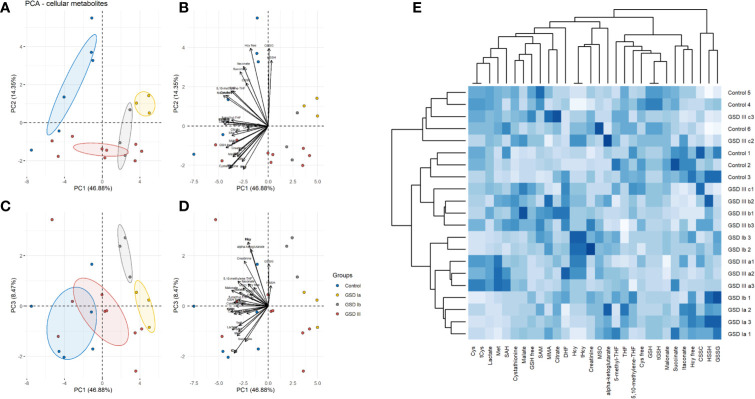
Principal component analysis (PCA) of intracellular metabolites. Panel **(A)** Score plots of PC1-PC2. Panel **(B)** Loading plots of PC1-PC2. Panel **(C)** Score plots of PC1–PC3. Panel **(D)** Loading plots of PC1-PC3. Control: n=3; GSDIa n =1; GSDIb n=1; GSDIII n=3, each by triplicate. A total of 31 metabolites were included in the analysis. Ellipses shown at 95% confidence centered around the mean of data points. The small overlap among ellipses depicts differences between healthy controls and disease subtypes. Panel **(E)** Heatmap of intracellular metabolites with hierarchical clustering (Euclidean distance, complete linkage).

The results of PCA for extracellular and intracellular metabolites are shown in **Figures 3** and **4**, respectively. The PCA score and loading plots for extracellular metabolites are shown in **Figures 3A–D**. The PCA score and loading plots for intracellular metabolites are shown in **Figures 4A–D**. As can be seen from both extracellular and intracellular samples, metabolite correlations determined distinct clustering of healthy controls versus GSD subtypes. A list of the individual contribution of metabolites to PC 1, PC 2, and PC 3 is provided in **Table 2**. Lactate, cysteine and cystathionine are common top contributors both in the intra- and extracellular compartments.

**Table 2:** Individual contribution of metabolites to variance in principal component analysis.

Intracellular metabolites	Individual contribution to variance (%)			Extracellular metabolites	Individual contribution to variance (%)		
	PC 1	PC 2	PC 3		PC 1	PC 2	PC 3
**GSH**	6.17	0.03	0.12	**Malate**	8.54	0.33	0.26
**tGSH**	6.17	0.03	0.12	**Citrate**	8.28	0.52	0.11
**Malonate**	5.92	2.25	0.92	**MSO**	8.02	2.30	1.17
**Lactate**	5.72	0.00	2.12	**Met**	7.82	2.39	4.76
**GSH free**	5.26	1.13	0.05	**DHF**	7.58	0.57	0.13
**Cys free**	5.17	2.30	0.22	**Succinate**	7.25	0.47	1.85
**Cys**	5.09	0.04	8.33	**Cystathionine**	7.19	1.30	4.44
**tCys**	5.09	0.04	8.33	**Lactate**	7.09	0.00	4.78
**Cystathionine**	4.58	4.40	0.00	**tCys**	6.41	4.64	4.79
**Met**	4.23	4.26	3.34	**5-methyl-THF**	5.90	3.61	0.01
5,10-methylene-THF	4.07	2.97	2.32	Creatinine	5.10	0.02	15.73
5-methyl-THF	3.97	0.08	0.15	tHcy	4.82	4.02	4.42
SAH	3.89	0.21	2.44	tGSH	4.65	0.04	8.43
SAM	3.84	0.74	0.24	SAH	3.91	3.90	16.05
THF	3.72	3.01	1.43	MMA	3.80	5.64	5.72
Malate	3.66	2.30	0.71	alpha-ketoglutarate	2.08	24.12	3.71
Citrate	3.20	0.09	0.00	SAM	0.58	31.45	0.33
MSO	2.80	2.24	1.23	Itaconate	0.58	14.50	23.21
Creatinine	2.54	1.46	7.63	Malonate	0.32	0.09	0.02
Succinate	2.33	7.16	4.75				
MMA	2.01	3.06	0.21				
Hcy	1.80	4.54	15.48				
tHcy	1.80	4.54	15.48				
Itaconate	1.79	8.61	1.83				
CSSC	1.44	4.50	0.55				
DHF	1.35	0.04	0.26				
alpha-ketoglutarate	1.33	0.00	12.09				
Hcy free	0.91	14.68	1.29				
HSSH	0.03	10.49	1.59				
GSSG	0.00	14.64	6.64				
Cummulative variance	100	100	100	Cummulative variance	100	100	100

Data are given for PC 1, PC 2, and PC 3, which are the major determinants of healthy control and disease subtype clustering.

GSH, glutathione; GSSG, oxidized glutathione; tGSH, total glutathione; Hcy, homocysteine; HSSH, homocystine; tHcy, total homocysteine; Cys, cysteine; CSSC, cystine; tCys, total cysteine; THF, tetrahydrofolate; DHF: dihydrofolate; MMA, methylmalonic acid; MSO, methionine sulfoxide; Met, methionine.

Metabolites marked in bold are the top 10 contributors’ variance on PC 1. Lactate, cysteine and cystathionine are common top contributors both in the intra- and extracellular compartments.

To further identify common and distinct features between healthy controls and GSD disease groups, we carried out hierarchical clustering on the metabolite data. GSD metabolites grouped together to a common cluster that is mostly separated from the healthy control group (**Figure 3E**: extracellular metabolites, **Figure 4E**: intracellular metabolites). Partial metabolite overlap was seen between healthy controls and GSDIII patient 3 (c2, c3), both for intracellular and extracellular components.

### Glycolysis and Krebs Cycle in GSD Fibroblasts

**Figure 5:**
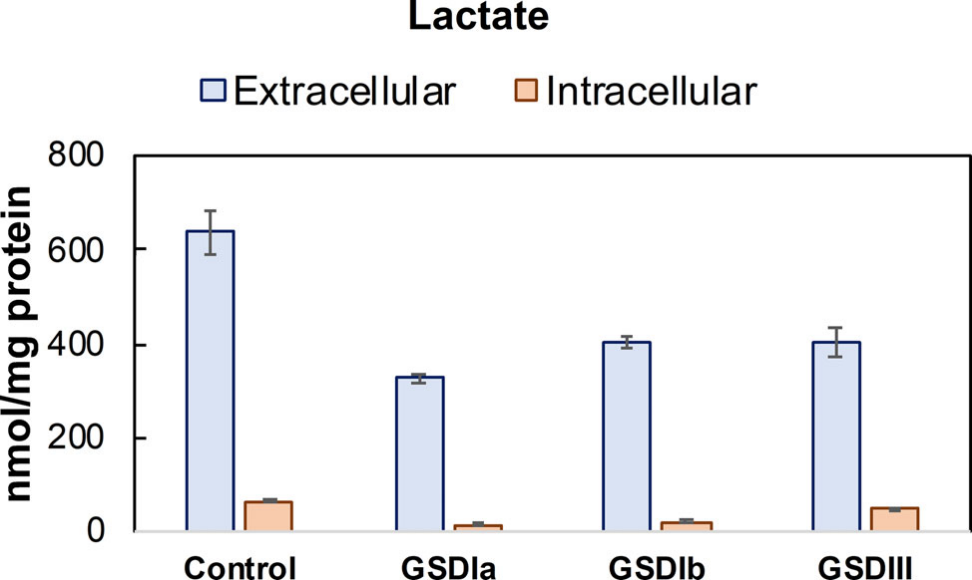
Lactate concentration in conditioned culture medium (extracellular) and cell lysates (intracellular). Lactate concentration determined in conditioned culture medium and cell lysates after 5 days in culture. Lactate concentration was determined by LC-MS/MS and normalized by total protein concentration. Control: n=2; GSDIa n =1; GSDIb n=1; GSDIII n=3, each by triplicate. Data are shown as mean ± standard deviation. Intracellular lactate was significantly reduced in GSDIa and GSDIb compared to healthy controls, in contrast to GSDIII that exhibited lactate concentration comparable to controls (individual comparisons, one-way ANOVA, p < 0.05). A comparison of extracellular lactate from healthy control cells versus all GSD cells did not result in statistically significant differences.

**Figure 6:**
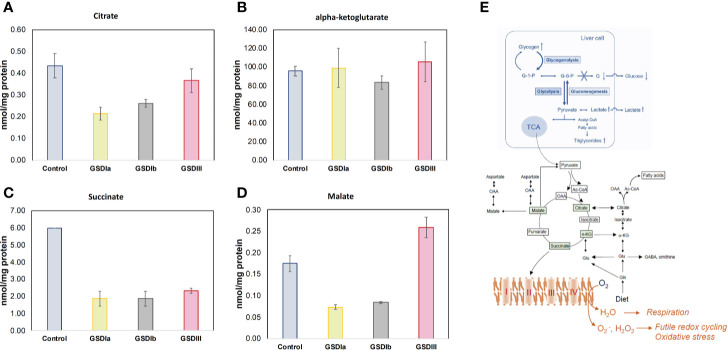
Concentration of Krebs cycle intermediates in cells. Panels **(A**–**D)** show citrate, alpha-ketoglutarate, succinate, and malate concentration, respectively, determined in cell lysates after 5 days in culture. Metabolite concentrations were determined by LC-MS/MS and normalized by total protein concentration. No statistically significant differences were found between healthy control and GSD cells for citrate or alpha-ketoglutarate, suggesting no impairments in incoming precursors of the Krebs cycle. The concentration of succinate was reduced in all GSD cells with respect to healthy control cells. The concentration of malate was lower in GSDIa and GSDIb compared to healthy controls, but no differences were found between healthy controls and GSDIII. Panel **(E)** summary of GSD enzymatic impairment highlighting routes of carbon unit flow into the Krebs cycle (TCA) and respiratory chain. All individual comparisons were performed with one-way ANOVA, p<0.05. Control: n=2; GSDIa n =1; GSDIb n=1; GSDIII n=3, each by triplicate. Data are shown as mean ± standard error of the mean.

Analysis of intracellular and extracellular lactate showed significant downregulation of glycolysis in all GSD subtypes compared to healthy controls (**Figure 5**). With this finding and the result of normal OCR in GSD fibroblasts, we reasoned that GSD cells may rely on oxidative phosphorylation more than glycolysis to sustain cellular energy demands under our experimental conditions. We therefore examined Krebs cycle intermediates citrate, alpha-ketoglutarate, succinate and malate (**Figure 6**). The concentration of citrate and alpha-ketoglutarate did not differ markedly between healthy controls and GSD fibroblasts, which indicates normal supply of precursor carbons into the Krebs cycle. Succinate, a substrate of complex II of the respiratory chain, was lower in all GSD fibroblasts in comparison to healthy controls, suggesting increased expenditure of this substrate into oxidative phosphorylation in the presence of disturbed glycogen storage. Lower concentration of malate was found in GSD Ia and GSD Ib and a greater concentration of this metabolite was present in GSD III with respect to healthy controls. This result suggests distinct demand of succinate into the respiratory chain to furnish mitochondrial respiration in these disease subtypes.

### Intracellular and Extracellular Creatinine in GSD Fibroblasts

**Figure 7:**
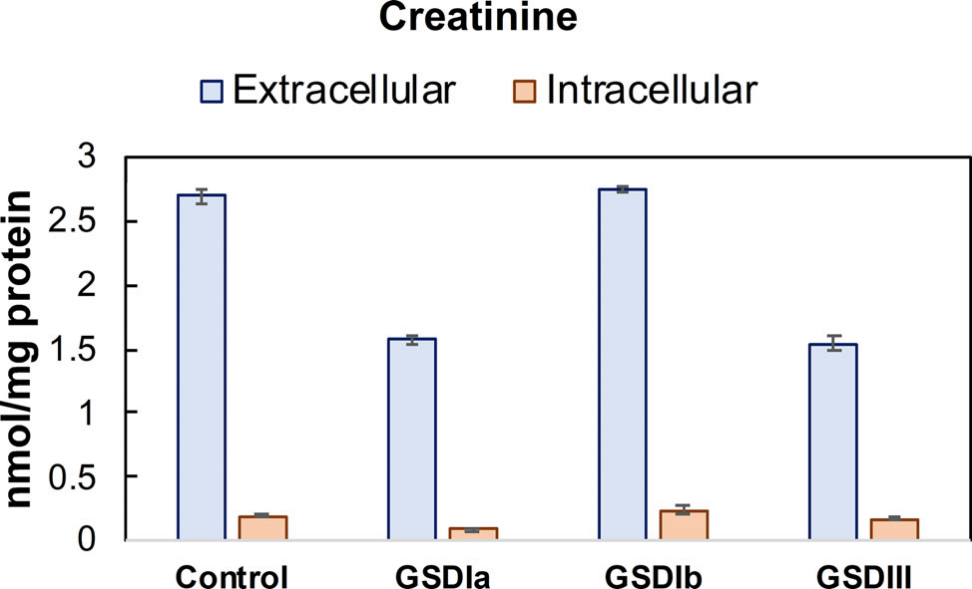
Concentration of creatinine in conditioned medium and cells. Creatinine concentration was determined in cell lysates after 5 days in culture. Metabolite concentrations were determined by LC-MS/MS and normalized by total protein concentration. Control: n=2; GSDIa n =1; GSDIb n=1; GSDIII n=3, each by triplicate. Data are shown as mean ± standard error of the mean.

Besides liver pathology, one of the physiological consequences of abnormal glycogen storage in GSD types I is renal injury (30–32). Analysis of extracellular creatinine (**Figure 7**) showed lower creatinine concentration in GSD Ia and GSD III compared to healthy controls, but statistically significance was only found for GSD III compared to healthy controls. The concentration of intracellular creatinine was comparable between healthy controls and all GSD subtypes (**Figure 7**). Noteworthy, within groups, mean extracellular creatinine mirrored the pattern of the respective intracellular concentrations, suggesting that maintenance of intracellular creatinine is partly controlled by export into the extracellular medium.

### Redox Homeostasis in GSD Fibroblasts: Cysteine and Glutathione Metabolism

**Figure 8:**
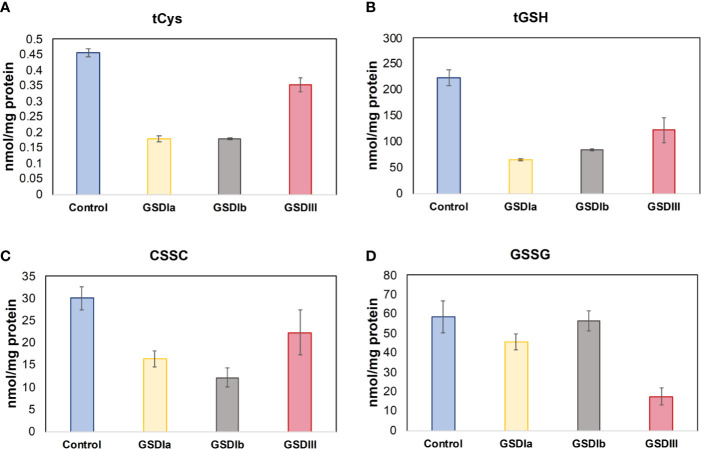
Status of cysteine and glutathione. Panel **(A)** shows the total concentration of reduced cysteine in cells after 5 days in culture. Panel **(B)** shows the total concentration of reduced glutathione in cells after 5 days in culture. Panel **(C)** Concentration of oxidized cysteine in cells after 5 days in culture. Panel **(D)** Concentration of oxidized glutathione in cells after 5 days in culture. Metabolite concentrations were determined by LC-MS/MS and normalized by total protein concentration. Individual comparisons of healthy controls versus GSDIa, GSDIb, and GSDIII showed significantly reduced concentration of Cys and GSH in all GSD cells compared to healthy controls. A reduced concentration of CSSC was found in all GSD cell lines compared to healthy controls. In contrast, GSSG concentration was comparable to healthy controls in GSDIa and GSDIb, and significantly reduced in GSDIII. Control: n=2; GSDIa n =1; GSDIb n=1; GSDIII n=3, each by triplicate. Data are shown as mean ± standard error of the mean. Statistical significance for individual comparisons was assessed with one-way ANOVA, p <0.05.

Insufficient control of glucose metabolism, with chronic hypo- and hyperglycemia as well as intermittent spikes of these extremes cause oxidative stress (33, 34). Because hypoglycemia is a characteristic of GSD Ia and GSD III (35), we examined thiol pools that represent the major low molecular weight redox buffers of the cell, reduced and oxidized glutathione (GSH/GSSG) and reduced and oxidized cysteine (Cys/CSSC). The Cys and GSH redox pairs act at the forefront of the cellular antioxidant defense. **Figure 8** shows the results of GSH and Cys pools in the intracellular compartment. Individual comparisons of healthy controls versus GSD Ia, GSD Ib and GSD III showed that all GSD cell lines exhibited significantly lower concentration of total Cys and GSH compared to healthy controls (p < 0.05, one-way ANOVA). In line with these findings, PCA also identified cystathionine, an element of the trans-sulfuration pathway that supports biosynthesis of cysteine and glutathione, as a major contributor to distinct metabolic phenotypes of healthy controls and GSD subtypes.

## Discussion

The objective of this study was to examine mitochondrial structure and function and metabolic profiles in human skin fibroblasts from GSD I and GSD III patients compared to healthy controls. The cohort of GSD fibroblasts selected in this study has been characterized by previous groups *via* genetic and enzymatic methods that confirmed impaired glycogen metabolism (**Table 1**) (10, 11, 14, 24). The cohort of GSD fibroblasts utilized is commercially available for research purposes thus permitting comparisons across methods and laboratories.

Herein, we sought out to investigate GSD in human cultured cells under conditions that do not pose metabolic stress. Cells were grown in low-glucose DMEM medium supplemented with 10% FBS, a composition that supports cellular proliferation by providing complete sources of carbohydrates, fatty acids and amino acids. Our qualitative examination of mitochondrial network in live human skin fibroblasts by confocal microscopy did not identify alterations in GSD cells versus healthy controls. Likewise, measurement of total mitochondrial volume and oxygen consumption rates retrieved no statistically significant differences between healthy control and GSD fibroblasts. We reasoned that GSD cells may operate at a metabolic poise that enables cell function at the expense of shifts in homeostatic metabolite concentrations that support energy metabolism. We therefore examined metabolites that are relevant to GSD phenotypes (lactate, creatinine), energy production (Krebs cycle), and redox homeostasis (Cys, GSH and related sulfur-containing metabolites) among others. A total of 20 extracellular and 31 intracellular metabolites were measured in this study. PCA of metabolites enabled the clustering of control and GSD groups, which was further observed after HC of these datasets. Intracellular metabolite profiles provided a clearer grouping between healthy controls and GSD subtypes compared to extracellular metabolites. These findings reveal for the first time, that the cellular and extracellular metabolic compartments of GSD subtypes differ from that of healthy controls in humans.

Analysis of metabolites that were ranked as major contributors to the first three principal components of PCA showed that the major elements impacted by GSD-causing mutations are glycolysis, Krebs cycle at the point of succinate, the entry substrate of mitochondrial respiratory complex II, and the status of the major intracellular antioxidant thiols cysteine and glutathione. This metabolic combination suggests increased demands for energy production with concomitant generation of reactive species that exhaust the antioxidant Cys and GSH systems, a scenario that reminisces futile redox cycling, i.e. electron leakage from the respiratory chain (**Figure 6E**). Thus, while the overall network and content of mitochondria in GSD appeared to be intact under our experimental conditions and there were no changes in basal oxygen consumption rates, our metabolite patterns recapitulate functional changes that are in line with mitochondrial dysfunction. Isotopic tracing of key metabolites of glycolysis (glucose-6-phosphate, glucose-1-phosphate), fatty acid oxidation, and amino acid catabolism together with the determination of cellular energy reserve in the form of AMP, ADP and ATP are essential to elucidate the precise adjustments in energy metabolism that occur in patients with glycogen storage disease.

Measurements of creatinine revealed reduced export in GSD Ia and GSD III fibroblasts compared to healthy controls. This suggests that impaired glycogen storage (deriving from a breakdown impairment) reduces the transport of creatinine out of the cell. Besides its previous identification in fibroblasts (36) and its housekeeping role in cellular energy metabolism, the consequences of reduced creatinine export on the onset and progression of renal disease in GSD patients remains to be investigated in an organ-relevant cell type, such as renal proximal tubular epithelial cells.

In sum, our study serves as proof-of-concept to further profile metabolism in GSD patients in cells, tissue and biological fluids. Gaining insights into the metabolic adjustments that occur upon disrupted glycogen metabolism may permit the dissection of profound consequences of pathogenic mutations that manifest essentially in all cell types, such is seen in fibroblasts, versus derangements that are specific to and modifiable in disease-relevant organs only, which could therefore be targeted for therapeutic purposes. Furthermore, access to quantitative metabolomics tools to investigate the response of cells and biological fluids to dietary management and current therapies opens new opportunities for personalized and optimized treatment of the complex and heterogeneous manifestations observed in GSD patients. In addition, this tool may be of great value to distinguish milder and severe disease phenotypes.

### Limitations of Study

Use of fibroblasts as cell type. While the usefulness of fibroblasts to study metabolic disorders is substantiated by the wide conservation of all pathways of energy metabolism in this cell type, and more specifically, of preserved glycogen storage phenotypes in early studies performed with human fibroblasts from GSD patients (14), further studies in cells representing the major affected organs (liver, muscle, kidney) are desirable and necessary. The human version of organ-specific cells are less accessibly for research purposes, which is highlighted by the existence of very few such studies, with the majority of findings deriving from animal models.
Size of cohort. This study examined one patient with GSD Ia, one patient with GSD Ib and three patients with GSD III. The small sample size of the study serves only as proof-of-concept to examine the metabolic behavior of the different impairments of glycogen storage metabolism in humans. Further studies with greater number of cells per clinical condition are necessary to validate our findings and extend conclusions at the population level.
Cell culture conditions. Experimental cell culture conditions do not perfectly represent dietary exposure and glycemic control of GSD patients. Further analysis of metabolomic features in cultured cells that more closely mimic nutritional and physiologically relevant glycemic control in GSD are currently underway, using our herein established experimental conditions as a starting point.
Targeted versus discovery “omics”. Our targeted metabolic profiling focused on known and predicted alterations of disrupted glycogen storage. It is possible that other metabolic pathways not covered in this study also contribute to pathogenesis for which untargeted “omics” approaches are desirable.

## Conclusion

The investigation of defective glycogen storage in human skin fibroblasts recapitulated features consistent with, albeit not identical to, mitochondrial dysfunction identified in animal models of the disease. The lack of structural defects in the mitochondria of GSD human skin fibroblasts may be related to organ specificity: previous studies performed in animal models examined the organelle in hepatocytes, representing the major organ affected by hepatic GSDs. Our findings together with results from the literature imply that systemic consequences of monogenic diseases of metabolism may have distinct impact on different cell types and organs, a proposal that is substantiated by the clinical manifestations of the disorders. Our studies identified metabolite features consistent with dysfunctional electron transport through the respiratory chain and dampened concentrations of cysteine and glutathione, the major low molecular weight antioxidants of the cell suggestive of increased oxidative stress. This proof-of-concept study opens opportunities for larger cohort studies with GSD patients where cells, tissue biopsies and biological fluids could be examined jointly to reconstruct the metabolic landscape of the disease and to possibly refine dietary and pharmacological treatment of the patients.

## Data Availability Statement

The raw data supporting the conclusions of this article will be made available by the authors, without undue reservation.

## Ethics Statement

Ethical review and approval was not required for the study on human participants in accordance with the local legislation and institutional requirements. Written informed consent for participation was not required for this study in accordance with the national legislation and the institutional requirements.

## Author Contributions

LH, SG, and US conceived and planned the study. JT, VW, KK, IB, and LH performed experiments. LH supervised experimental work. LH, SG, IB, RN and US performed data analysis and wrote the manuscript. All authors contributed to the article and approved the submitted version.

## Funding

This work was funded with intramural support from the Department for Pediatrics, Center for Metabolism, Faculty of Medicine, Medical Center, University of Freiburg. SG acknowledges a research funding donation from Nutricia Metabolics. The article processing charge was funded by the Baden-Wuerttemberg Ministry of Science, Research and Art and the University of Freiburg in the funding programme Open Access Publishing.

## Conflict of Interest

The authors declare that the research was conducted in the absence of any commercial or financial relationships that could be construed as a potential conflict of interest.
