# Supplementary material for: Metabolic Profiling in Human Fibroblasts Enables Subtype Clustering in Glycogen Storage Disease
Source: Front Endocrinol (Lausanne). 2020 Nov 23;11:579981. doi: 10.3389/fendo.2020.579981 (PMC7719825; doi:10.3389/fendo.2020.579981)
Supplement: Supplementary file 1 [file DataSheet_1.pdf]

Supporting Information for:

Metabolic profiling in human fibroblasts enables subtype clustering in glycogen storage disease

Luciana Hannibal[1]*, Jule Theimer[1], Victoria Wingert[1], Katharina Klotz[1], Iris Bierschenk[2], Roland Nitschke[2,3], Ute Spiekerkoetter[4] and Sarah C. Grünert[4]*.

[1]Laboratory of Clinical Biochemistry and Metabolism, Department of General Pediatrics, Adolescent Medicine and Neonatology, Faculty of Medicine, Medical Center - University of Freiburg, 79106 Freiburg, Germany

[2]Life Imaging Center, Center for Integrated Signalling Analysis, Albert-Ludwigs University, Freiburg, 79104, Germany

[3]BIOSS Centre for Biological Signaling Studies, Albert-Ludwigs-University Freiburg, Freiburg, 79104, Germany

[4]Department of General Pediatrics, Adolescent Medicine and Neonatology, Faculty of Medicine, Medical Center - University of Freiburg, 79106 Freiburg, Germany

Correspondence: luciana.hannibal@uniklinik-freiburg.de and sarah.gruenert@uniklinik-freiburg.de

Table S1. Composition of DMEM, low glucose, GlutaMAX™, pyruvate (Gibco product 21885; Thermofisher product Nr. 21885025, 21885108) utilized to grow human healthy control and GSD skin fibroblasts.

Figure S1. Oxygen consumption rate of healthy control and GSD fibroblasts.

Figure S2. Lactate concentration in conditioned culture medium.

Figure S3. Imaging of mitochondria in fibroblasts from healthy controls and from patients with GSD subtypes Ia, Ib and III.

Table S1. Composition of DMEM, low glucose, GlutaMAX™, pyruvate (Gibco product 21885; Thermofisher product Nr. 21885025, 21885108) utilized to grow human healthy control and GSD skin fibroblasts.

Components	Molecular Weight	Concentration (mg/L)	mM
**Amino Acids**			
Glycine	75.0	30.0	0.4
L-Alanyl-Glutamine	217.0	862.0	3.9723501
L-Arginine hydrochloride	211.0	84.0	0.39810428
L-Cystine	313.0	48.0	0.15335463
L-Histidine hydrochloride-H2O	210.0	42.0	0.2
L-Isoleucine	131.0	105.0	0.8015267
L-Leucine	131.0	105.0	0.8015267
L-Lysine hydrochloride	183.0	146.0	0.7978142

L-Methionine	149.0	30.0	0.20134228
L-Phenylalanine	165.0	66.0	0.4
L-Serine	105.0	42.0	0.4
L-Threonine	119.0	95.0	0.79831934
L-Tryptophan	204.0	16.0	0.078431375
L-Tyrosine	181.0	72.0	0.39779004
L-Valine	117.0	94.0	0.8034188
**Vitamins**			
Choline chloride	140.0	4.0	0.028571429
D-Calcium pantothenate	477.0	4.0	0.008385744
Folic Acid	441.0	4.0	0.009070295
Niacinamide	122.0	4.0	0.032786883
Pyridoxine hydrochloride	206.0	4.0	0.019417476
Riboflavin	376.0	0.4	0.0010638298
Thiamine hydrochloride	337.0	4.0	0.011869436
i-Inositol	180.0	7.2	0.04
**Inorganic Salts**			
Calcium Chloride ($CaCl_2$-$2H_2O$)	147.0	264.0	1.7959183
Ferric Nitrate ($Fe(NO_3)_3$ $9H_2O$)	404.0	0.1	2.4752476E-4
Magnesium Sulfate ($MgSO_4$-$7H_2O$)	246.0	200.0	0.8130081
Potassium Chloride (KCl)	75.0	400.0	5.3333335
Sodium Bicarbonate ($NaHCO_3$)	84.0	3700.0	44.04762
Sodium Chloride (NaCl)	58.0	6400.0	110.344826
Sodium Phosphate monobasic (NaH_2PO_4-$2H_2O$)	156.0	141.0	0.90384614
**Other Components**			
D-Glucose (Dextrose)	180.0	1000.0	5.5555553
Phenol Red	376.4	15.0	0.039851222
Sodium Pyruvate	110.0	110.0	1.0

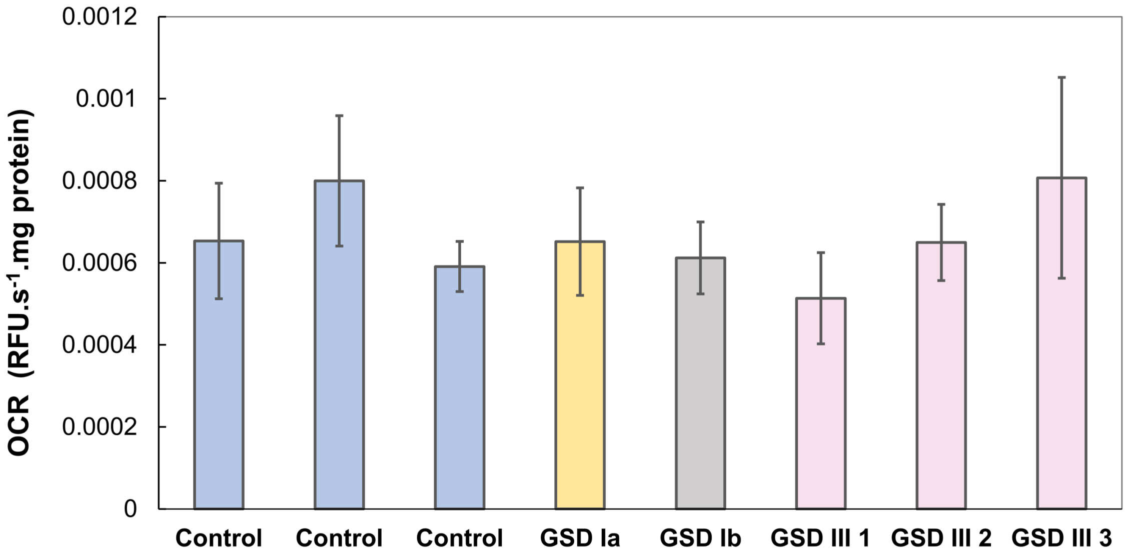

Figure S1. Oxygen consumption rate of healthy control and GSD fibroblasts. Oxygen consumption rates were normalized by performing cell lysis and total protein quantification immediately after data collection. Control: n=3; GSDIa n =1; GSDIb n=1; GSDIII n=3, each by triplicate. Data are shown as mean ± standard deviation.

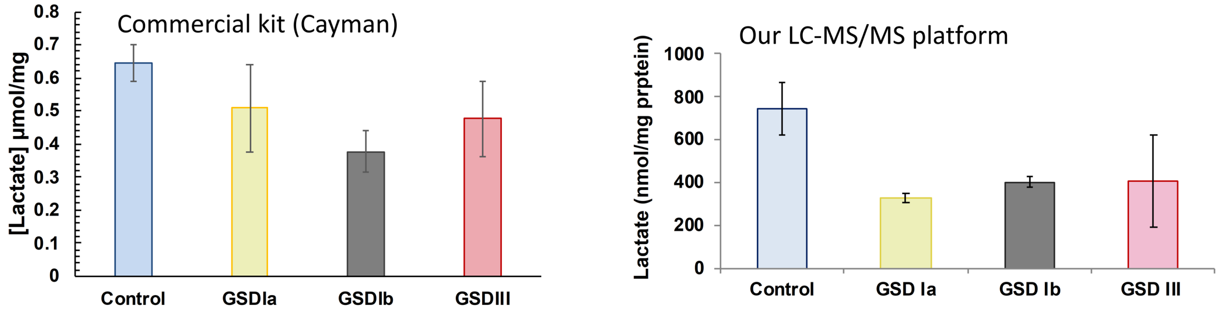

Figure S2. Lactate concentration in conditioned culture medium. Panel A: lactate concentration measured using a colorimetric reaction that uses lactate dehydrogenase (Cayman). Panel B: Lactate concentration measured by LC-MS/MS. Data are shown as mean ± standard deviation.

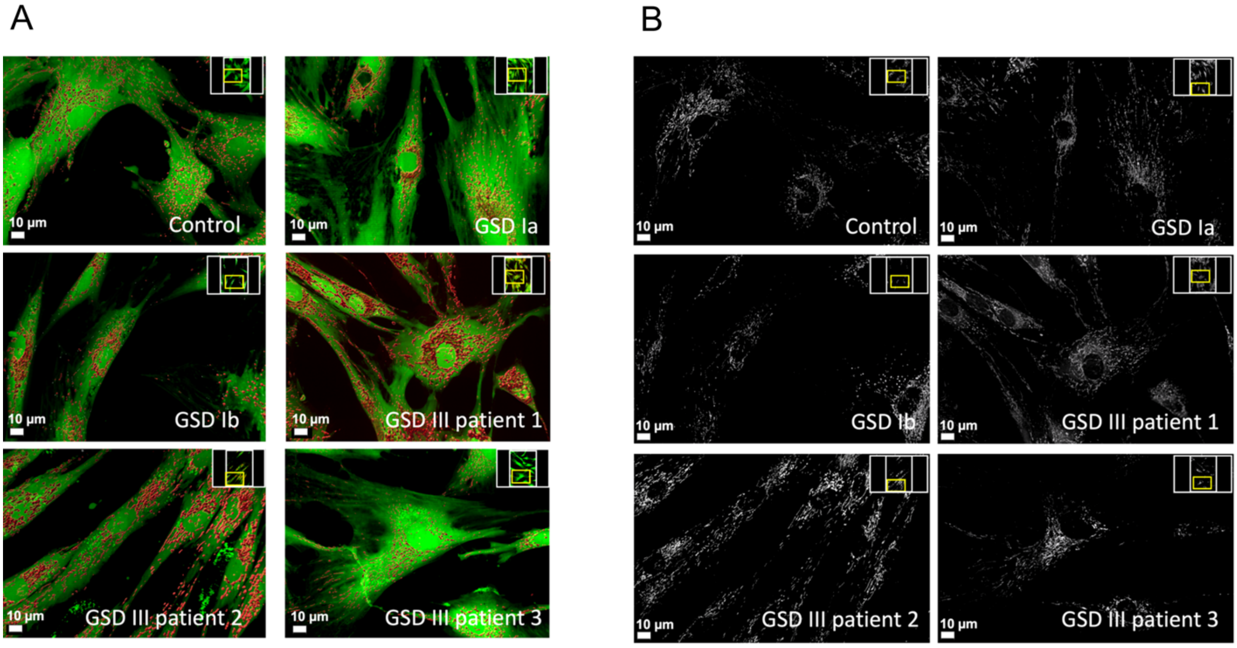

Figure S3. Imaging of mitochondria in fibroblasts from healthy controls and from patients with GSD subtypes Ia, Ib and III. Panel A. Visualization of rendered mitochondrial volumes (red) overlaid upon cell body (green) surfaces. Images processed with Imaris software. Panel B. Respective visualization of mitochondria in single z-plane greyscale images picked from the same data sets as in Panel A. Scale bars on the lower left corner represent 10 µm. In the upper right image corners the full image recording area is shown used for the analysis data shown in Fig. 2 of the manuscript and with the region marked selected for the magnified image area.
